# Deviations in early hippocampus development contribute to visual hallucinations in schizophrenia

**DOI:** 10.1038/s41398-020-0779-9

**Published:** 2020-03-25

**Authors:** Arnaud Cachia, Claire Cury, Jérôme Brunelin, Marion Plaze, Christine Delmaire, Catherine Oppenheim, François Medjkane, Pierre Thomas, Renaud Jardri

**Affiliations:** 1 Université de Paris, Institut de Psychiatrie et Neurosciences de Paris, INSERM, GHU Paris psychiatrie & neurosciences, F-75005 Paris, France; 2 Université de Paris, Laboratoire de Psychologie du développement et de l’Education de l’Enfant, CNRS, F-75005 Paris, France; 3 Institut Universitaire de France, Paris, France; 4 Department of Medical Physics and Biomedical Engineering, University College, London, UK; 5 Univ Rennes, CNRS, Inria, Inserm, IRISA UMR 6074, EMPENN — ERL U 1228, F-35000 Rennes, France; 6 INSERM U 1028, CNRS UMR-5292, Lyon Neuroscience Research Center, PSYR2 Team, Université de Lyon, CH le Vinatier, Lyon, France; 7 CHU Lille, Salengro Hospital, Neuroradiology dpt, 59000 Lille, France; 8 CHU Lille, Hôpital Fontan, Plateforme CIC - CURE, 59000 Lille, France; 9 Univ Lille, INSERM U-1172, CHU Lille, Lille Neuroscience & Cognition Centre (LiNC), Plasticity & SubjectivitY (PSY) team, 59000 Lille, France

**Keywords:** Physiology, Hippocampus

## Abstract

Auditory hallucinations (AHs) are certainly the most emblematic experiences in schizophrenia, but visual hallucinations (VHs) are also commonly observed in this developmental psychiatric disorder. Notably, several studies have suggested a possible relationship between the clinical variability in hallucinations′ phenomenology and differences in brain development/maturation. In schizophrenia, impairments of the hippocampus, a medial temporal structure involved in mnesic and neuroplastic processes, have been repeatedly associated with hallucinations, particularly in the visual modality. However, the possible neurodevelopmental origin of hippocampal impairments in VHs has never been directly investigated. A classic marker of early atypical hippocampal development is incomplete hippocampal inversion (IHI). In this study, we compared IHI patterns in healthy volunteers, and two subgroups of carefully selected schizophrenia patients experiencing frequent hallucinations: (a) those with pure AHs and (b) those with audio–visual hallucinations (A+VH). We found that VHs were associated with a specific IHI pattern. Schizophrenia patients with A+VH exhibited flatter left hippocampi than patients with pure AHs or healthy controls. This result first confirms that the greater clinical impairment observed in A+VH patients may relate to an increased neurodevelopmental weight in this subpopulation. More importantly, these findings bring crucial hints to better specify the sensitivity period of A+VH-related IHI during early brain development.

## Introduction

Hallucinations—erroneous perceptions that are not elicited by external stimuli—may manifest in every sensory modality^1^. In schizophrenia, auditory hallucinations (AHs) have been described as the dominant experiences, with occurrence rates ranging from 60% to 80%^2^. Although visual hallucinations (VHs) have been largely neglected in psychiatric disorders, a systematic review showed evidence of a weighted mean of 27% of VHs in schizophrenia^3^. In contrast to what can be observed in neurological or eye diseases, schizophrenia is characterized by very rare isolated VHs^4^, which typically co-occur with hallucinations in other sensory modalities^5–9^, notably with auditory hallucinations in up to 84% (later called “A+VH”^10^).

Impairments of the hippocampus, a medial temporal structure involved in mnesic and neuroplastic processes, have been repeatedly reported in schizophrenia. Such changes include reductions in volume, increases in basal perfusion, activation deficits during declarative memory, and reductions in neurogenesis in the dentate gyrus (for a recent review, see ref. ^11^). More precisely, hippocampal hyperactivity was regularly associated with auditory^12,13^, visual^14^, or multisensory hallucinations^15^. Beyond a strict local alteration, disrupted hippocampal oscillations were linked to functional changes in hallucinations-related network^16^, while we observed specific structural and functional hippocampal dysconnectivity patterns in patients with audio–visual hallucinations^17^.

The fact that some schizophrenia patients experience pure AH or A+VH experiences has been related to developmental factors. Several studies reported that the rate of VHs in schizophrenia was age-dependent^18–20^, and some authors proposed that they could be considered as a severity index of developmental abnormalities^19^, a theory in line with the neurodevelopmental model of schizophrenia, which considers this disorder as the end state of abnormal brain development starting years before its onset^21^. This hypothesis was recently supported by the association of VHs with impaired cortical sulcation^22^, an indirect proxy of early deviations in brain development^23,24^.

Surprisingly, the question of a possible neurodevelopmental origin of hippocampal impairments in VHs has never been investigated. A classic marker of early atypical hippocampal development is incomplete hippocampal inversion (IHI)^25–27^. IHI is a variant of the hippocampus anatomy, in which prominent features are round, verticalized, and medially positioned hippocampus^28,29^. Different terms have been used to refer to this atypical pattern, including “hippocampal malrotation”^30–32^, “abnormal hippocampal formation”^29^, or “developmental changes of the hippocampal formation”^28^.

IHI has been described in patients with seizures (with a prevalence of ~30–50%), particularly in the case of impaired cortical development and in temporal lobe epilepsy^28,29,33,34^. However, IHI is not specific to epilepsy and has also been reported in healthy individuals, although with a lower frequency^25,29,35^. A recent study assessed the prevalence of IHI in the general population in a large sample of over 2000 subjects and reported more frequent IHIs in the left (17%) than in the right (6%) hemispheres^36^.

In this context, this study aimed to test the hypothesis of a neurodevelopmental hippocampal deviation specifically associated with VHs in schizophrenia. We compared healthy volunteers with two subgroups of carefully selected seizure-free schizophrenia patients experiencing frequent hallucinations: patients with pure AH (i.e., patients who had never reported visual hallucinations) and patients with A+VH. The matched subgroups of patients differed only in the presence or absence of VHs. This distinction appears crucial in testing for variable IHI in patients with hallucinations according to the sensory modality involved.

## Materials and methods

### Participants

Forty-six right-handed participants were included in the study, including 30 outpatients suffering from schizophrenia and 16 healthy controls (HCs) with no personal history of psychiatric disorder or family history of psychosis. Of the 30 patients, there were 16 AH patients and 14 A+VH patients. None of the patients reported hallucinations in another sensory modality. All patients met the DSM-IV-TR criteria for schizophrenia based on interviews and review of their clinical history by an experienced psychiatrist. The Positive and Negative Syndrome Scale (PANSS)^37^ and the Scale for the Assessment of Positive Symptoms (SAPS)^38^ were used to evaluate general psychopathology and to quantify symptom severity. All patients received these semistructured interviews, which included a detailed assessment of their lifetime hallucinatory experiences. All patients were noted to have marked-to-severe auditory hallucinations (SAPS-it. #1 ≥ 4). Patients from the AH group had never experienced visual hallucinations (i.e., SAPS-it. #6 = 0), whereas A+VH patients scored greater than 4 on the SAPS-it. #6.

All subjects were otherwise medically healthy and reported no history of seizure, head trauma, other neurological disease, or significant current major medical conditions based on history and medical examination. None of the patients reported substance abuse, with the exception of four patients reporting the occasional consumption of cannabis (two in the AH group and two in the A+VH group). No patient with an IQ below 80 was included. Groups were matched for age and sex (all *p* > 0.7); AH and A+VH patient groups were also matched for symptom severity, including auditory hallucinations and antipsychotic dosage (Table 1). Group matching for age and sex notably allows controlling for potential confounding effects on IHI. All patients were treated with antipsychotic medications at the time of the study (atypical antipsychotics *n* = 29, typical antipsychotics *n* = 4). Olanzapine-equivalent daily doses were calculated in reference to recent international guidelines to assess the homogeneity of antipsychotic dosages across groups^39^. The study was approved by the local ethics committee (CPP Nord-Ouest IV, France). Written documentation of informed consent and the capacity to provide consent was obtained from each participant prior to enrollment. Clinical data analyses are summarized in Table 1.

**Table 1:** Demographical and clinical characteristics of the 49 participants enrolled in the study: 33 patients with schizophrenia based on the presence of auditory only (AH) or audio–visual hallucinations (A+VH), and 16 healthy controls (HC).

	Healthy control (HC)	Patients with auditory hallucinations (AH)	Patients with audio–visual hallucinations (A+VH)	AH vs A+VH (*p*-value)
Sample size	16	16	14	—
Sex (male/female)	10/6	10/6	8/6	0.9
Age (mean ± SD)	29.5 ± 9.9	30.4 ± 9.6	29.5 ± 10.2	0.8
PANSS score				
Total (mean ± SD)	—	76.7 ± 16.7	69.5 ± 20.1	0.3
Positive (mean ± SD)	—	19.0 ± 4.6	21.4 ± 5.6	0.2
Negative (mean ± SD)	—	20.4 ± 6.4	16.2 ± 7.7	0.1
General (mean ± SD)	—	37.3 ± 9.2	32.0 ± 11.1	0.15
SAPS score		30.5 ± 11.2	38.1 ± 13.5	0.1
Item 1 (mean ± SD)	—	4.4 ± 0.5	4.58 ± 0.6	0.4
Item 6 (mean ± SD)	—	0 ± 0.3	4.5 ± 0.4	<0.0001
Olanzapine-equivalent dose (mean ± SD)	—	22.8 ± 10.6	19.2 ± 11.3	0.4

Quantitative (resp. qualitative) demographic and clinical characteristics comparisons between groups were based on bilateral Student’s *t* (resp. Chi^2^) tests.

### MRI acquisition and processing

All participants underwent a 10-min anatomical T1-weighted sequence (3D multi-shot turbo-field-echo scan; 150 transverse slices, field of view = 256 mm², and voxel size = 1 mm^3^) on a 1.5T Intera Achieva scanner (Philips, Netherlands). All subjects wore headphones and earplugs to attenuate the noise of the scanner. These MRI parameters were considered adapted to the analysis of individual hippocampal morphologies.

To perform IHI assessment with a standardized orientation, T1-weighted MRIs were registered to the MNI152 atlas using FSL software with the fully automated affine transformation FLIRT^40,41^. All MRI data were anonymized, and manual labeling of IHIs was carried out blind to the participant’s demographic, clinical characteristics, and group attribution.

### Classification of hippocampal patterns

**Fig. 1:**
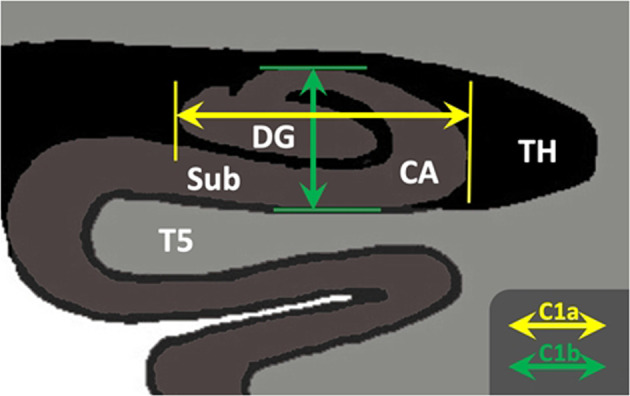
Anatomical criteria used to define hippocampal roundness in a coronal MRI view. The horizontal arrow (C1a) goes from the medial part of the dentate gyrus (DG) to the lateral part of the hippocampus. The vertical arrow (C1b) goes from the bottom to the top part of the cornu Ammonis (CA). When C1a > C1b, the hippocampus is considered “flat”, and round/oval otherwise (later called “nonflat”). Sb subiculum, TH temporal horn.

Hippocampal patterns were classified based on the main IHI criteria^25,30,31,42^: roundness, sometimes referred to as “pyramidal shape”. Hippocampal roundness was evaluated in coronal slices on the first half of the hippocampal body as detailed by Cury et al.^36^ (see Fig. 1). Two segments, C1a and C1b, were visually determined. Segment C1a represents the width of the hippocampus in a coronal view. It is parallel to the ventral part of the cornu ammonis (CA), which is next to the subiculum and extends from the medial part of the dentate gyrus to the lateral part of the CA. Segment C1b represents the height of the hippocampal body in a coronal view. C1b is perpendicular to segment C1a and goes from the dorsal part of the hippocampus to the ventral part of the CA.

Roundness was categorized into two levels: “flat” (width larger than height, i.e., C1a > C1b) or “nonflat” (i.e., round or oval: C1a ≤ C1b). Intra- and interobserver reproducibility of these hippocampal roundness categories, previously estimated in an independent sample of 900 subjects^36^, revealed strong agreement (kappa = 0.7).

### Statistical analyses

Between-group differences in IHI distribution (“flat” vs. “nonflat”) in the left and right hemispheres were analyzed using binomial generalized linear models (GLM) with sex (“male” vs “female”) and group (“A+VH” vs. “AH” vs. “HC”) as categorical factors and age as a quantitative covariate. When a significant main or interactive effect involving groups was detected, analysis was followed by post hoc analyses. The main effects and interactions were probed with the Chi-squared test. A two-tailed *p*-value < 0.05 was considered statistically significant. All statistical analyses were carried out with R 3.4.3 software (http://www.r-project.org/) and the “car”, “effects”, and “nnet” libraries.

## Results

**Fig. 2:**
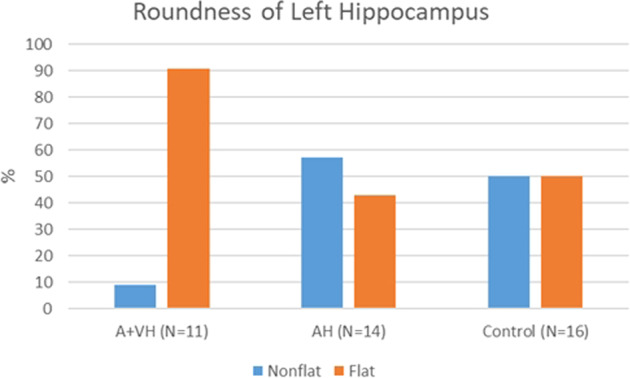
Frequency distribution of incomplete hippocampal inversions (IHIs) in the three experimental groups, based on hippocampal roundness (color-coded). Schizophrenia patients with audio–visual hallucinations (A+VH, left) exhibit significantly flatter left hippocampus patterns than schizophrenia patients with pure auditory hallucinations (AH, middle) or matched healthy controls (right).

Table 2 and Fig. 2 summarize the frequency distribution of IHI observed in the HCs and in each subgroup of schizophrenia patients (i.e., with AH or A+VH). Due to poor MRI contrast, IHI could not be evaluated in the left or right hemispheres of 5 participants.

**Table 2:** Hippocampal patterns (hippocampal roundness) in healthy controls (HC, *N* = 16), patients with auditory only (AH, *N* = 16), and patients with audio–visual (A + VH, *N* = 14) hallucinations.

	HC	AH	A + VH
*Left hippocampus*			
Nonflat % (*N*)	50 (8)	57 (8)	9 (1)
Flat % (*N*)	50 (8)	43 (6)	91 (10)
*Right hippocampus*			
Nonflat % (*N*)	20 (3)	21 (3)	18 (2)
Flat % (*N*)	80 (12)	79 (11)	82 (9)

Generalized linear model analysis revealed a significant main effect of group on left hippocampus roundness (Chi^**2**^ = 6.536, *p* = 0.038), but not on the right (Chi^2^ = 0.239, *p* = 0.887). The main effects for age and sex on left or right roundness were not significant. Post hoc analyses indicated a specific distribution of left roundness patterns in A+VH patients, who exhibited significantly flatter left hippocampus patterns than AH patients (Chi^2^ = 7.864, *p* = 0.005) and the HCs (Chi^2^ = 4.028, *p* = 0.044). No distribution difference was detected between the HCs and AH patients (Chi^2^ = 0.281, *p* = 0.595).

## Discussion

Both postmortem and in vivo studies suggest a pivotal role of hippocampal formation in the pathophysiology of schizophrenia in general^11^, and of complex hallucinations in particular^14,17^. However, the early developmental deviations that may contribute to hippocampal impairments in A+VH have never been directly investigated. This study was designed to specifically address this issue by comparing IHI, a marker of early hippocampal development, between healthy controls, schizophrenia patients with pure AH, and those who experience A+VH.

We found that VHs were associated with a specific IHI pattern, independent of the underlying diagnosis (i.e., schizophrenia), or the presence of unisensory hallucinations (i.e., AHs), two features shared by the patient groups. We notably demonstrated that schizophrenia patients with A+VH exhibited flatter left hippocampi than patients with pure AH or healthy controls. This result is fully in line with previous research stating that the greater clinical impairment and greater compromise of overall functioning observed in A+VH patients may relate to an increased neurodevelopmental weight in this subpopulation^19,22^. More importantly, these findings bring crucial hints to better specify the sensitivity period of A+VH-related IHI during early brain development.

The hippocampus is the first cortical area to differentiate during fetal life^43^, and most of the features observed in the adult population are acquired by gestational week (GW) 30. Primordial hippocampi seem to be observable from GW 7^44^. During the rotational growth of the telencephalic vesicle, the major portion of the hippocampus is carried dorso-laterally, and then ventrally to lie in the medial aspect of the temporal lobe. As the neocortex expands and evolves, the allocortex is displaced inferiorly, medially, and internally into the temporal horn^44^. These various developmental processes mainly drive hippocampus inversion, classically probed by hippocampus roundness^25,30,31,42^.

Interestingly, hippocampal roundness was also shown to reflect brain immaturity. In preterm neonates, an IHI is observed in 50% of neonates aged between 23 and 24 GWs, in 24% of neonates aged between 25 and 28 GWs, and in 14% of neonates aged between 29 and 36 GWs^45^. This pattern allows us to infer that the high proportion of A+VH patients found with a flat left hippocampus likely reflects an early neurodevelopmental vulnerability to VHs. This interpretation supports and extends previous findings in A+VH patients found to have abnormal sulcation^22^, another marker of prenatal brain deviation^24^. Similar associations between specific psychotic features and decreased sulcation were also reported^46,47^, but the present IHI findings provide the first evidence that the vulnerability period for A+VH precedes 23 GWs, an earlier window than the 25–29 GWs previously proposed for AHs^47^.

Neurodevelopmental vulnerability during fetal life does not exclude the effects of later stressors during postnatal development. First, animal studies already showed that factors altering early postnatal hippocampal neurogenesis are essential in schizophrenia-like symptoms progression as a whole^48^. Second, human studies have shown that early-life insults before 5 years, but not later in childhood, can be responsible for the association between stress severity and reduced hippocampal volumes^49^. Interestingly, childhood trauma has regularly been reported to be associated with the severity of AH in psychosis (e.g. ref. ^50^) or the sensory complexity of hallucinations in youth^51^, while higher scores in the Childhood Trauma Questionnaire (CTQ) were found to be correlated with increased resting hippocampal perfusion in individuals at ultrahigh risk for psychosis^52^, a pattern also linked with AH occurrences^13,15^.

Another major finding of this study concerns laterality issues in VH since structural brain asymmetries are determined during fetal life (e.g. ref. ^53^). We know that asymmetric development of the hippocampus is common and that IHI is more frequent in the left hemisphere than in the right hemisphere^36^. Furthermore, unilateral right IHI is particularly rare^26,33^. From a functional point of view, the right hippocampus is predominantly involved in location memory, whereas the left hippocampus plays a central role in context-dependent episodic or autobiographical memory^54–56^, two cognitive functions that have also been proposed to be involved in hallucinations (e.g. refs. ^57,58^).

Crucially, hyperactivation of the left hippocampus has been evidenced during AHs^13^ but also prior to the emergence of the hallucinatory state^12,59^, while inputs from the left hippocampal complex to the salience network have been shown concomitant to complex hallucinations’ occurrences^15^. The left hippocampus was thus proposed to trigger memory fragments and bring them to consciousness, causing intrusive percepts (e.g. ref. ^60^). Even if this is beyond the scope of this paper, we can note some similarities between this theory of hallucinations and other paroxistic neural activities also linked with IHI, such as epilepsy. Together, these findings support our assumption that (i) hippocampal inversion can be incomplete, mainly in the left hemisphere, if this process is stopped at a specific time during development and that (ii) the left hippocampus is involved in pathological phasic processes, such as hallucinations.

Two potential shortcomings need to be acknowledged. First, the sample size used in this experiment was moderate. This issue is compensated by a high subgroup homogeneity, which allowed us to address strong a priori hypotheses and draw significant conclusions. The comparison of subgroups of patients with or without VHs allowed for the assessment of the specific effects of the hallucinatory modality and complemented the more conventional comparison between schizophrenia patients and HCs (the specific effects of VHs must be distinguished from disease- or AH-related effects). Furthermore, because patients with multisensory hallucinations are often described as more severe, we were vigilant to avoid any confounds linked with global severity by matching the two patients subgroups on PANSS scores. Finally, because IHI is frequent in patients with epilepsy, we ensured that none of the enrolled participants had a history of epilepsy. Second, visual inspections of hippocampal formation could be considered a potential replicability issue. However, the good agreement of our method with automated identification was previously established^36^, making us confident in the reliability of our findings.

Overall, this study supports the involvement of at least two types of neurodevelopmental factors in A+VH. While previous lines of evidence support a link between changes in quantitative features of brain anatomy, such as cortical thickness or volume, and AHs (e.g. ref. ^61^), we were able to show how qualitative features, such as IHI, could give insight into deviations that occur prenatally and pinpoint a vulnerability to more complex hallucinatory experiences. The quantitative features were shown to be more sensitive to specific interactions, even early in life, such as childhood trauma, but IHI is the only able to reflect very early developmental vulnerability to A+VH, well before the onset of hallucinations or even schizophrenia and its prodrome. More precisely, we showed that A+VH exhibits a first sensitivity period, which likely precedes the 23rd gestational week. If replicated, such findings would support the development of targeted stress prevention/protection interventions intended to at-risk pregnant women.

Precise mechanisms leading to IHI and A+VH are still to be deciphered. Because the placenta was recently shown to modulate the genomic risk for schizophrenia^62^, it could be interesting in future studies to specify which psychological and biological factors from the intrauterine environment are able to impact the trajectory of the brain and the later risk for AH and A+VH. Future studies could also explore the possible association between IHI and performances in cognitive functions regulated by specific hippocampal subfields^63^, such as contextual binding^58^ or source memory^64^.
